# Herbal Medicines for Inflammatory Diseases 2016

**DOI:** 10.1155/2016/8270323

**Published:** 2016-10-25

**Authors:** Seong-Gyu Ko, Chang Shik Yin, Bing Du, Kyoung Hyun Kim

**Affiliations:** ^1^Laboratory of Clinical Biology and Pharmacogenomics and Center for Clinical Research and Genomics, College of Korean Medicine, Kyung Hee University, Seoul 02447, Republic of Korea; ^2^Acupuncture and Meridian Science Research Center, College of Korean Medicine, Kyung Hee University, Seoul 02447, Republic of Korea; ^3^Shanghai Key Laboratory of Regulatory Biology, School of Life Science, Institute of Biomedical Sciences, East China Normal University, Shanghai, China; ^4^Division of Environmental Genetics and Molecular Toxicology, Department of Environmental Health, College of Medicine, University of Cincinnati, Cincinnati, OH 45267, USA

 Inflammation is, in a sense, a way of life. Inflammation is both protective and destructive to the body. Balance and the process of balance maintenance matter in inflammation. On the one hand, inflammation has been discussed as a critical factor in inflammatory diseases like rheumatoid arthritis and asthma. Inflammatory process is also important in such conditions as cancer, obesity, and stroke. On the other hand, inflammatory process is an essential protective mechanism for limiting the range of tissue damage and thereafter process of tissue repair. That was why we have called inflammation as a two-faced Janus in the editorial of the special issue 2014.

Inflammation has long been recognized in the history of medicine. Since ancient days, inflammatory nature and typical symptoms and signs of many conditions have been identified, investigated, and treated. Although the degree of understanding and successful treatment on inflammatory conditions were limited, herbal medicine has almost always served as a rich and ample source of treatment agent for the inflammation. Modern bioscience and biomedicine are not an exception. Herbal medicine is an important source of new drugs and new treatments from research benches to bedsides. Herbal medicine is considered as both historic and modern. There remains much to be elucidated in relation with precise mechanisms and developed in relation with clinical effects of herbal medicines, which will have a vast impact on the understanding of the nature of inflammation and the health.

We are very pleased at the publication of this special issue. It was our pleasure and honor to receive precious manuscripts from renowned researchers and prepare the special issue on this invaluable topic. This special issue delivers indispensable and up-to-date research reports on the herbal medicines for inflammatory diseases. The source herbs and materials are from geographically and culturally diverse regions. The target conditions range from skin wound to dermatitis, colitis, mastitis, acute myocardial ischemia, periodontitis, stroke, asthmatic or inflammatory lung diseases, and obesity. Insightful reviews and interesting investigations in this special issue will be an important contribution to this unrelentingly growing field of research and will draw attentions from those who are eager to be a witness and participating member of the advancement of the researches on herbal medicines for inflammatory diseases, we hope.

*Seong-Gyu Ko* *Seong-Gyu Ko* *Chang Shik Yin* *Chang Shik Yin* *Bing Du* *Bing Du* *Kyoung Hyun Kim* *Kyoung Hyun Kim*

